# Identity and Gender Recognition Using a Capacitive Sensing Floor and Neural Networks

**DOI:** 10.3390/s22197206

**Published:** 2022-09-23

**Authors:** Daniel Konings, Fakhrul Alam, Nathaniel Faulkner, Calum de Jong

**Affiliations:** Department of Mechanical & Electrical Engineering (MEE), School of Food & Advanced Technology (SF&AT), Massey University, Auckland 0632, New Zealand

**Keywords:** biometrics, capacitive floor, gender classification, human sensing, machine learning, neural network

## Abstract

In recent publications, capacitive sensing floors have been shown to be able to localize individuals in an unobtrusive manner. This paper demonstrates that it might be possible to utilize the walking characteristics extracted from a capacitive floor to recognize subject and gender. Several neural network-based machine learning techniques are developed for recognizing the gender and identity of a target. These algorithms were trained and validated using a dataset constructed from the information captured from 23 subjects while walking, alone, on the sensing floor. A deep neural network comprising a Bi-directional Long Short-Term Memory (BLSTM) provided the most accurate identity performance, classifying individuals with an accuracy of 98.12% on the test data. On the other hand, a Convolutional Neural Network (CNN) was the most accurate for gender recognition, attaining an accuracy of 93.3%. The neural network-based algorithms are benchmarked against Support Vector Machine (SVM), which is a classifier used in many reported works for floor-based recognition tasks. The majority of the neural networks outperform SVM across all accuracy metrics.

## 1. Introduction

Subject recognition within the built environment has many applications and can facilitate Ambient-Assisted Living (AAL), emergency response, etc. Similarly, gender classification can allow for guided navigation in shopping malls for gender-targeted sales or security within single-sex dormitories. Another potential application of this could be in the retail sector. If the movement of customers in a shop can be separated based on gender, it may be possible to utilize that information for gender-specific product placement. There have been many reported works on gender/identity recognition using wearable sensors (e.g., [1]). However, subject intervention or occupant compliance render them impractical in many applications. Computer vision-based techniques [2] require clear line of sight and can be perceived as invasive to privacy in many scenarios. Likewise, acquiring physical biometric traits such as speech, fingerprints, facial features, etc. are often inherently intrusive by nature and typically require client initiation.

Every individual has a unique behavioral trait: their gait. Gait is becoming an increasingly popular biometric for health and aged care. For example, gait-related parameters can help identify risk factors for falls [3] and thus can lead to intervention (e.g., prevention through improving gait and mobility by exercises [4,5]). While wearable sensor [6] and computer vision-based gait analysis techniques [7,8] show good performance, they can be deemed as intrusive. The literature shows that it might be possible to extract gait information in an unobtrusive manner using floor-based sensing [9,10].

### 1.1. Floor-Based Sensing

As an individual walks on a floor, each footstep becomes the source of a physical excitation. This has been leveraged to identify and localize subjects and detect activities [11,12,13,14,15,16]. One of the benefits of such floor-based human sensing techniques is the potential to capture gait information.

Reported floor-based techniques employ pressure-sensitive floors [13,17], which are a network of seismic sensors to capture footstep-induced vibrations [14,15,16], etc. Pressure sensitive floors such as GaitRite [18] have been utilized in clinical settings for many gait-related studies. Unfortunately, such floors are not cost-effective for large-scale residential/non-clinical deployment. Pressure sensors are also not suitable for long-term deployments, as they are likely to degrade over time. Vibration-based techniques [12,19,20,21] have shown promising performance. However, significant challenges are yet to be resolved. Floors are a complex and heterogenous propagation medium (for vibration signals), and there is considerable variability from building to building. There have also been attempts utilizing acoustic techniques to identify subjects and gender by capturing the sound of footfall with microphones [22]. Unfortunately, the accuracy of such microphone-based approaches is low. Table 1 provides a summary of subject and gender recognition works that utilized floor-based sensing. One of the major limitations of all the techniques is the relatively low number of subjects the algorithms have been trained and tested upon. It should be noted that Vera-Rodriguez et al. [23] and Costilla-Reyes et al. [24] have performed footstep recognition with a large number of subjects (40 and 120, respectively). Unfortunately, they only capture a single stride, right foot followed by the left, of a subject on two small sensing mats with a large number of embedded piezo-electric sensors to capture pressure magnitude. This, in our opinion, is insufficient to capture important gait parameters such as cadence, cycle time, speed and even natural stride length.

#### Capacitive Sensing

Capacitive sensing functions by detecting changes in the capacitive coupling between tracked targets and custom sensors embedded within the surrounding walls [25] or flooring [26]. In floor-based implementations, the presence of tracked targets feet acts as a capacitive plate, coupling with a floor-embedded sensor to form a capacitor. As the target moves between embedded sensors, the target alters the electric field across the capacitors, creating measurable differences in the capacitance between sensors. These embedded floor capacitors can be implemented in various different ways including sewing wires in serpentine [27] or triangular forms [28] into a textile; using two sets of parallel wires, orthogonal to each other [29]; or metal squares [30]. In contrast to the aforementioned methods which use the human body as one plate of the capacitor (loading mode of capacitive sensing [31]), TileTrack [32] employs the transmit mode of capacitive sensing by emitting a signal from the floor which is continuously read by an additional electrode acting as a receiver. The change in signal amplitude between the transmitting floor and receiving electrode due to a roaming human presence is used to infer a target’s location.

Early literature on capacitive human sensing largely focused on improving sensing resolution; vehicle safety applications; or as a human–machine interface. In recent years, floor-based approaches have become more prevalent in the literature, as advances in signal processing and the introduction of machine learning have allowed improved feature detection and the identification of behavior on a per-user basis.

Fukui et al. [33] utilized capacitive flooring to detect walking activity but did not differentiate users. Contigiani et al. [34] utilize a mechanical foot apparatus and no test subjects. Siegmund et al. [35] attempted to reduce tailgating through security checkpoints by detecting people closely transiting through an entryway. Shi et al. [36,37] used Convolutional Neural Networks with triboelectric capacitive sensors embedded within the floor to identify either groups of people or individuals with accuracies varying between 85 and 96%. Li et al. [38] used triboelectric sensors to measure gait features, allowing eight individuals to be classified with an accuracy of 97.6% using a BLSTM network. An issue with this capacitive approach is that it requires pressure-based floor deformation to operate, introducing fatigue-based longevity concerns similar to pressure-based sensor implementations. Hoffmann et al. [9] explored gait mode classification using a capacitive sensing floor and an LSTM network, Other measurement methods have been used to detect: gender [39,40]; gait on steps [41]; emotional, height, and criminal detection [42]; fatigue [43]; identity [44]; and footsteps [21,45] and spatio-temporal gait parameters [46]. However, these approaches either required the subject to be tagged with a device, required the detection of floor vibration which would vary as the flooring aged, or suffered from low accuracy.

### 1.2. Contribution

We recently introduced, CapLoc [26], a prototype capacitive floor that can accurately localize a subject and also has the potential to detect falls by capturing fall poses. In this paper, we extend CapLoc’s capability by developing a data-driven, machine learning approach for subject and gender recognition. This work offers the following novel contributions:

- We rigorously benchmark several neural network structures for identifying individuals by using capacitive sensing data. We demonstrate that the Bi-directional Long Short-Term Memory (BLSTM)-based algorithm is the most accurate for subject identification, attaining an accuracy of 98.12%.
- To the best of our knowledge, this is the first reported work on gender recognition using capacitive floors. Among the several neural networks employed, Convolutional Neural Net (CNN) was found to be the most accurate for recognizing subjects’ biological gender with an accuracy of 93.3%.
- We have utilized more test subjects than previous works to provide a more robust generalization across varying subjects, while attaining high classification accuracy. This addresses a major limitation of the state of the art.

The rest of the paper is organized as follows. Section 2 discusses the physical implementation of the system. Section 2.1 presents the experimental data collection. Section 2.2 presents neural network development for gender and individual classification. Section 3 provides the performance of the benchmarked networks, and Section 4 concludes the paper.

## 2. Materials and Methods

**Figure 1:**
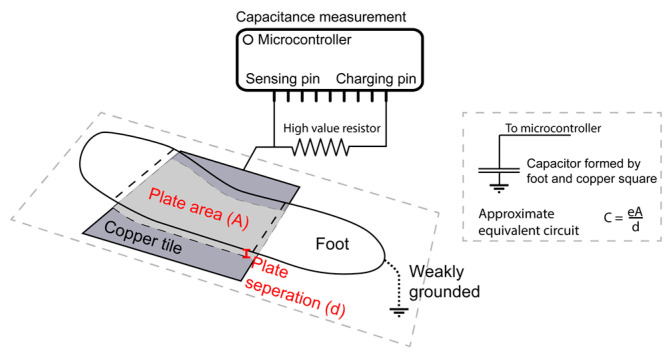
Loading mode capacitor formed by a subject’s foot.

The work utilizes a flooring prototype that has an array of capacitive sensors embedded underneath. We recruited twenty-three subjects to walk on the prototype floor. As subjects walk over the floor, a 200-pixel grayscale representation is obtained with a 10 Hz update rate. The capacitance readings from the floor are recorded from each participant traversing the area with 10 repeats. A portion of these data is used to train, validate and optimize ten neural network-based classifiers and two Support Vector Machines, which are used to recognize either the identity or the gender of each subject. The accuracy of the classifiers is tested on the remainder of the data and reported using various standard metrics. The floor, CapLoc [26], is based on the sensing changes in loading mode capacitance [31], where the sensors form one plate of a capacitor, with a target’s foot forming the other plate. This can be modeled as:

(1) $$C_{i}=\varepsilon \frac{A}{d}$$

where $C_{i}$ is the capacitance of the *i*th sensing plate, ε is the permittivity of the dielectric, *A* is the overlapping area between the plates and *d* is the distance between the plates. Assuming the floor is rigid, and each capacitive sensor is the same, ε and d remain constant, with *A* changing for affected sensors based on foot placement, as shown in Figure 1.

**Figure 2:**
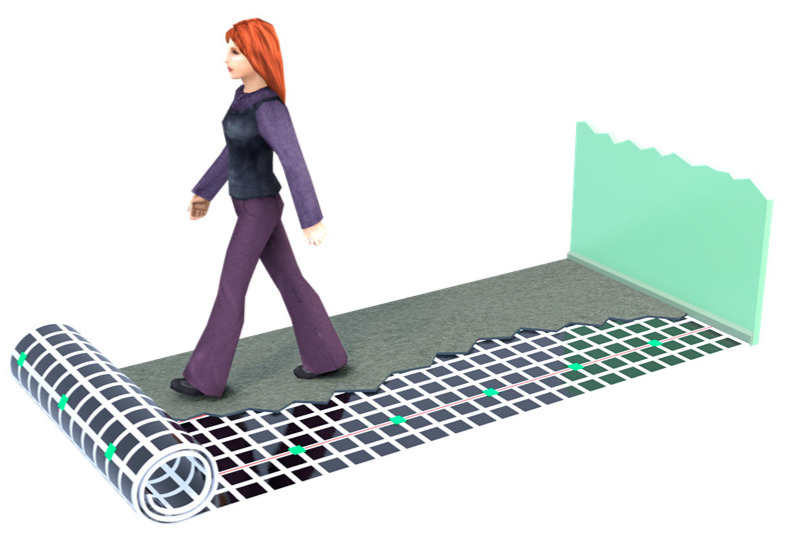
The structure of the capacitive sensing floor. Any non-conductive flooring can be laid on top of the sensing bed.

CapLoc is constructed from 0.6 m × 0.6 m sensing panels, each containing 25 copper squares used to form capacitor plates. Multiple sensing panels can be joined to form a floor, as shown in Figure 2. The data extraction from the floor is facilitated by its modular design.

**Figure 3:**
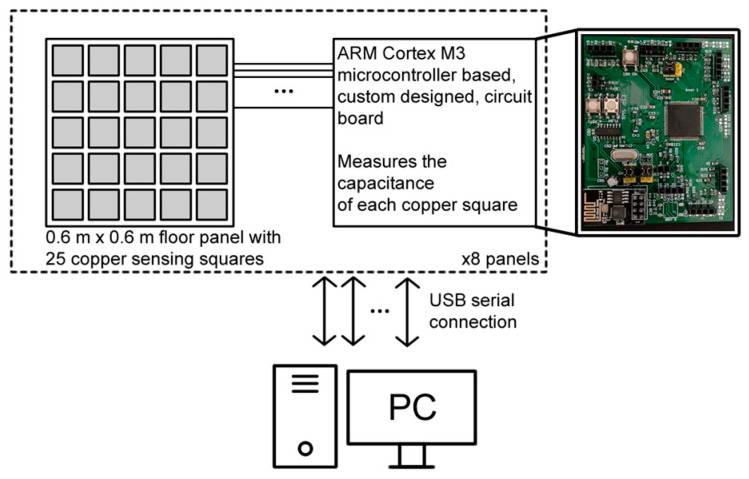
System architecture showcasing the pc–board–panel connections.

**Figure 4:**
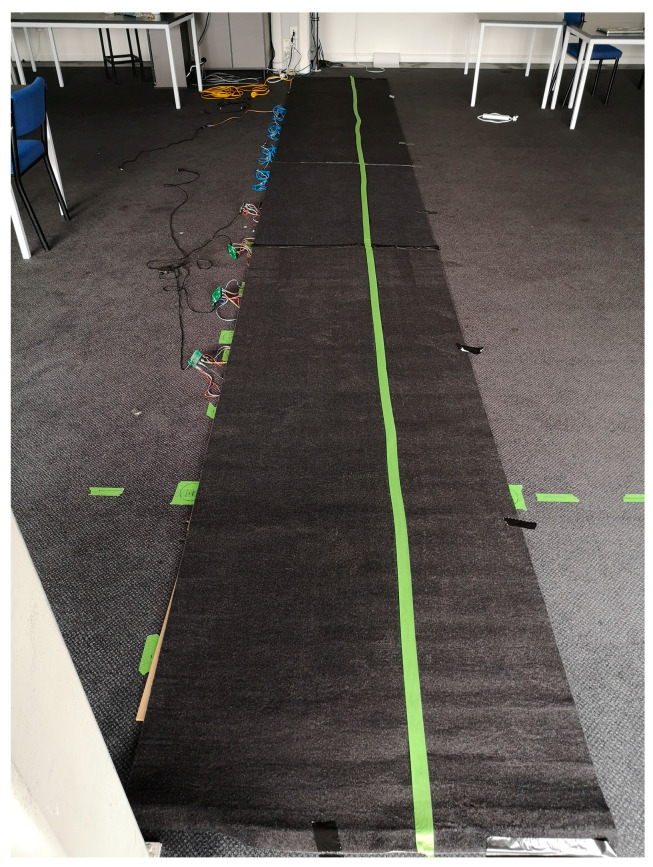
Eight sensing floor panels with carpet installed above forms the testbed the subjects walked on.

If a walking subject’s feet are considered to be weakly grounded, we can measure the time taken for the capacitor to charge to a set voltage $V_{0}$, using the *RC* time constant and then use this to gauge an estimate of a squares current capacitance using:

(2) $$V\left(t\right)=V_{0}\left(1-e^{-t/RC_{i}}\right)$$

where resistance *R* is selected as a sufficiently high value (>500 KOhm) so that it can be assumed to be constant, and it is independent of the foot’s unknown resistance to ground. Each panel uses a dedicated ARM cortex M3 microcontroller to sample the 25 readings at 10 Hz and sends them to a PC over serial, as shown in Figure 3. Please refer to Faulkner et al. [26] for more details of the capacitive sensing floor. Eight sensing panels were joined to form a test floor of 0.6 m × 4.8 m, as shown in Figure 4. The development work to scale up the prototype to cover a large room is currently ongoing. Onboard processing at the microprocessor is being investigated to lower the data rate and make the flooring more scalable.

### 2.1. Data Collection

**Figure 5:**
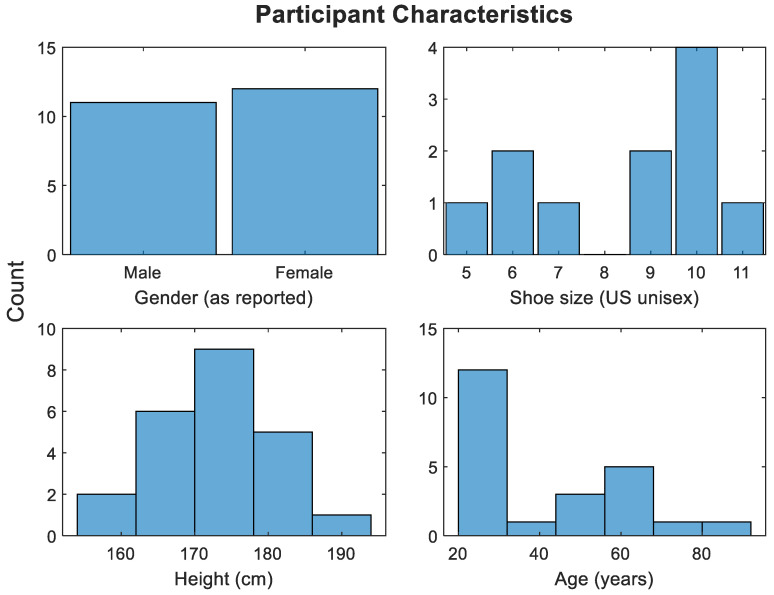
Self-reported participant characteristics.

This project involved the collection of walking data from 23 participants who all gave informed consent. Due to our ethics approval, we excluded: participants under 16, anyone with mobility injuries, anyone who could not give written consent, and anyone who used any form of walking assist (cane, etc.). The participant characteristics are shown in Figure 5. Please note that while weight was not recorded, it was not used as an exclusion criterion, as we wanted to recruit participants of a wide range of body types. Participants were requested to walk upon a linear 7.2 m walkway. The walkway consists of eight contiguous sensing panels covered by carpet and “dummy sections” to provide entry and exit from the sensing floor without having to change the walking pattern on the sensing panels. The carpet covers the walkway and is securely taped to the floor of the testing room. The edges of the floor bed are marked by a green tape line that participants can visibly see in their walking. An examiner with a laptop starts and finishes recording of the walking sequence for the participants, keeps track of the number of repeats, and assigns each subject’s recordings with a unique numerical identifier, noting whether they were male or female. Ten repeats were required of each participant.

Participants were instructed to keep their head forward and walk in a normal manner so as to record a natural gait pattern. Participants completed the trials at their own pace, with no restrictions given to walking speed. Participants were kept separate during testing so that they did not observe the walking behavior of any other individual across the sensing floor. The subjects were offered the chance to walk the trial area a few times before the recording starts to familiarize themselves with it. Enclosed running or walking shoes were required as a suggestion by the literature.

### 2.2. Machine Learning Approaches

The floor essentially creates a grayscale image of 200 pixels. Through continuous sampling of the floor, it captures 10 such images every second. Since the capacitance is formed when the subject’s foot meets the floor, unique temporal and spatial features of a subject’s gait (e.g., cadence, stride length, the angle of foot placement with respect to the direction of travel, the sequence of heel strike, foot plant and toe push off etc.) are being captured. We hypothesize that machine-learning classifiers can be trained to extract these (and potentially other) gait features to identify the gender of a subject along with their identity. Machine learning approaches are well equipped to extract features and classify data into bins when a true class is known. The subject identification was posed as a supervised multiclass classification problem that follows a standard model training followed by inference. The gender recognition is a supervised binary classification problem (each walking run is labeled with a number representing who walked across the floor and a second label with gender) [47]. We included only two genders (cisgender male and cisgender female), as we found it difficult to sign up subjects who are gender diverse. In order to make sure that the classifier is trained without bias, the training corpus should contain an equal number of each class. Unfortunately, we were only able to recruit cisgender volunteers (a recent survey indicates 99.2% of New Zealand’s adult population are cisgender [48]). Therefore, the gender classifier was trained as a binary classifier and as of now can only identify cisgender males and cisgender females.

In the literature, SVM is the most common classifier used for floor-based subject recognition. We therefore used a multiclass SVM with trained hyperparameters as our benchmark alongside against the neural network approaches. CNN and LSTM-based algorithms have been proposed in the subject identification literature (see Table 1) and they were, in fact, selected for our work based on the findings from the literature review. However, floor-based approaches vary significantly based on the technology implementation. This means that algorithms cannot be directly compared, as optimal hyperparameters vary based on the underlying data. To ensure fairness with our collected data, we ran an automated Bayesian hyperparameter optimizer to tune the parameters of the neural networks within our search space. This ensures that the accuracy of each approach is the highest within its search space, enabling fair algorithm comparison.

To explore whether different neural network architectures offer a competitive advantage over SVM for capacitive floor sensing, a traditional Multi-Layer Perceptron (MLP) structure was trained alongside a Convolutional Neural Network (CNN) and several recurrent neural networks: Long-Short Term Memory (LSTM), Bi-directional LSTM (BLSTM), and Gated Recurrent Units (GRU). CNN was included as the testbed containing eight 5 × 5 sensing panels can be assumed to have spatial correlation, as when both feet are touching the ground, the distance between them and the toe-in/toe-out walking gait could help uniquely identify users. The recurrent structures were chosen as they have the potential to exploit time-based features as a subject traverses an area. Three structures were chosen which can each offer potential benefits during training. The GRU structure has the benefit of fewer parameters for each layer compared to an LSTM, allowing them to potentially need less training data to generalize well. BLSTM was also chosen to explore whether feeding walking behavior in both forwards and backwards can contribute to stronger identifiable features for classifying identity or gender. Finally, since Support Vector Machines (SVM) have performed well in the literature (as shown in Table 1), a multiclass SVM was implemented for identity recognition, with a binary SVM used for gender recognition benchmarks. Since floor-based approaches vary significantly based on the technology implementation, this means that algorithms cannot be directly compared, as optimal hyperparameters vary based on the underlying data. To ensure fairness with our collected data, we ran an automated Bayesian hyperparameter optimizer to tune the parameters of the neural networks within our search space. This ensures that the accuracy of each approach is the highest within its search space, enabling fair algorithm comparison. The optimizer chose 23 × one-vs.-all binary classifiers with linear kernels, BoxConstraint of 0.04175, and KernelScale of 18.332 for the Identity classifier. The optimizer chose a one-vs.-all binary classifier with a linear kernel, BoxConstraint of 0.017556, and KernelScale of 0.96796 for the Gender classifier. In this work, a baseline reading of the floor’s capacitance was taken, which was subtracted from all subsequent readings, with the resulting output normalized between 0 and 1 before feeding it to the neural networks for training. For MLP networks, the 5 × 5 × 8 data frame was flattened to a 200 × 1 vector for training. For BLSTM, LSTM, and GRU networks, each timestep of the data stream was then flattened into 200 × 1 vectors before packaging vectors into one second long sequences for training. For the CNN network, the data were input as a single channel image 5 × 40 × 1, representing the eight 5 × 5 panels concatenated lengthwise.

For each machine learning approach, a Bayesian optimizer was used to tune the hyperparameters shown in Table 2 and Table 3 to attain the best performance. This was completed by using the first 7 recordings for each person to train a network for each set of hyperparameters before using the 8th recording set to calculate the validation accuracy. Once the networks were trained, the 9th and 10th recording sets were used for test data to report the final accuracy of each model, as given in Table 4 and Table 5. The performance metrics are defined as follows:

(3) $$Precision=\frac{\#\;TP}{\#\;TP+\#\;FP}$$

(4) $$Recall=\frac{\#\;TP}{\#\;TP+\#\;FN}$$

(5) $$Accuracy=\frac{\#\;Correct\;Classifications}{\#\;Total\;Classifications\;}$$

(6) $$F1\;Score=2\frac{Precision*Recall}{Precision+Recall}$$

where # represents ‘Number of’, *TP* represents ‘True Positives’ and *FN* represents ‘False Negatives’.

## 3. Results

**Figure 6:**
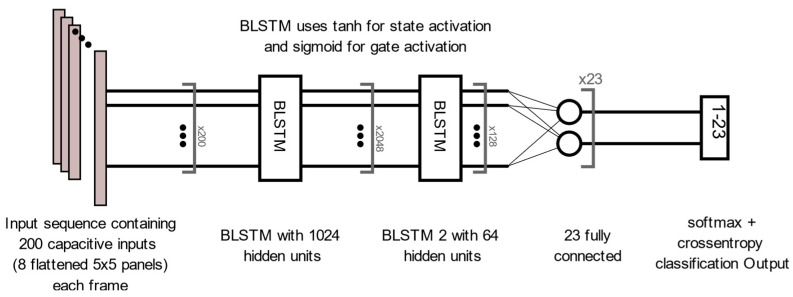
Best Performing Identity Classification Structure: BLSTM.

**Figure 7:**
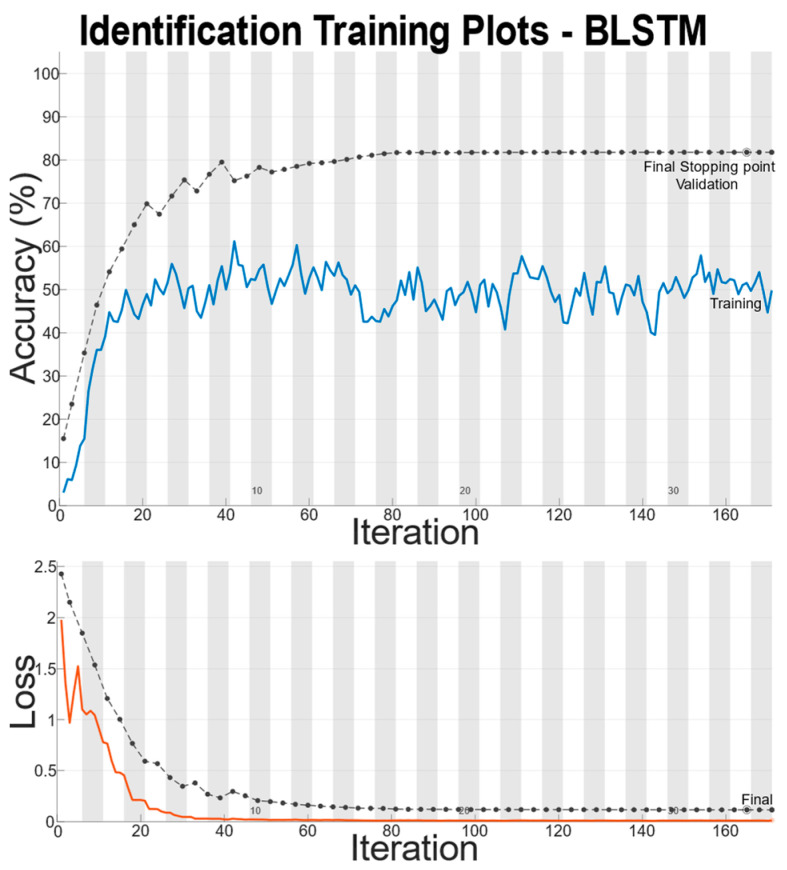
Training Plot for Best Performing Identity Network.

**Figure 8:**
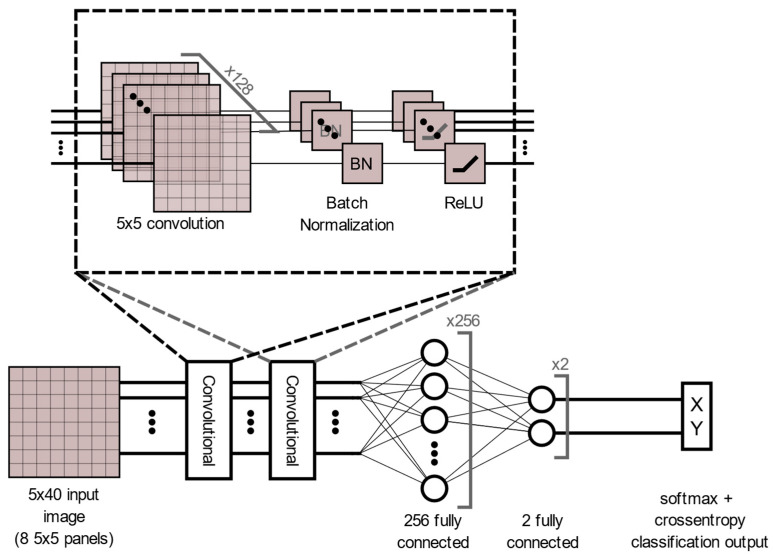
Best Performing Gender Classification Structure: CNN.

**Figure 9:**
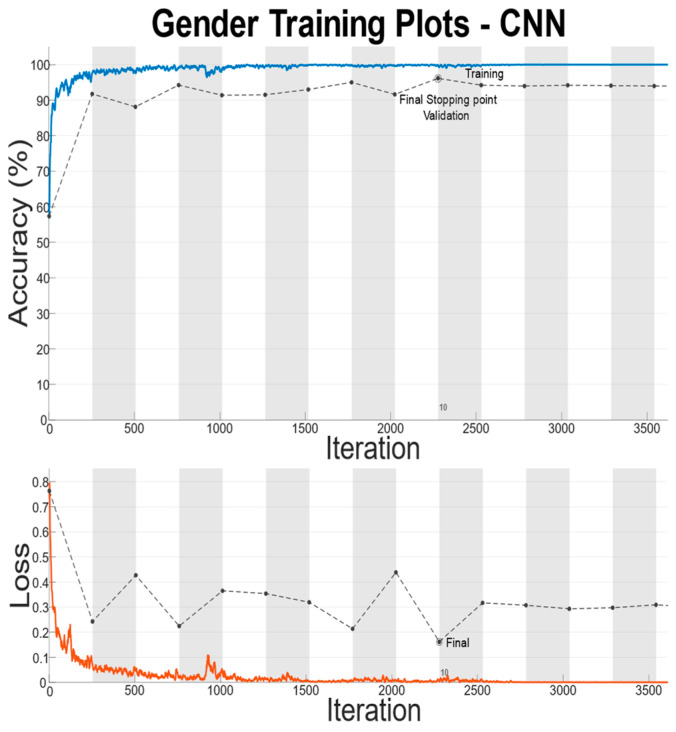
Training Plots for Best Performing Gender Network.

There is a large disparity in the complexity of the trained models, with a learnable parameter difference of almost 45× for the identity models and 11× for the gender models. This clearly shows a difference in the models’ ability to attain an abstract representation of the underlying features. All neural networks outperformed the baseline SVM in identity classification, with only the BLSTM underperforming the baseline SVM in gender recognition. Of interest was that while MLP networks did not offer best performance in either identity or gender classification tasks, their performance was not significantly lagging the leading approaches, and they had the best balance of performance to learnable parameters. This suggests that if models need to be deployed on resource-constrained edge computing devices (battery or memory), a traditional MLP-based approach may offer adequate accuracy while minimizing network size. Recurrent approaches such as LSTM and BLSTM provided the best performance for identity recognition, with BLSTM accurately identifying the subject 98.12% of the time. The network structure and training plots of the best performing BLSTM identity network are shown in Figure 6 and Figure 7 respectively. We believe that this may be due to BLSTM’s ability to extract temporal features from both forward/reverse passes of each trial, enabling finer-grained feature extraction of each subject’s gait. In contrast, the CNN network exploiting spatial features provided the best accuracy for gender recognition at 93.3%. The network structure and training plots of the best-performing CNN gender network are shown in Figure 8 and Figure 9, respectively. It should be noted, however, that the hyperparameter optimized CNN networks required more learnables for both identity and gender models than other networks, making them less suitable for deployment on resource-constrained devices. An interesting takeaway is that while temporal information was useful in identifying who a subject was, it did not lead to superior gender identification in our testing. This suggests that spatial features present within a static image seen by the CNN (shoe size, step length, pronation/supination, heading, etc.) may have a stronger correlation with gender than temporal features such as walking speed, hip swing, foot drag, etc.

When analyzing the identity model, it is important to explore whether any of the errors in classification can be attributed to common traits among users. Consider subject 16 who is misclassified as subject 15 16.6% of times, resulting in the lowest classification accuracy. These two subjects are of the same gender, with a height difference of 2 cm and age difference of 1 year. Their shoe sizes differ by 2. While this may suggest that the error arises from some obvious similarity between the pair, it should be noted that subject 15 is not misclassified as subject 16. She is rather misclassified (1.4%) as subject 13 who while being within 2 years of age is of a different gender, 16 cm taller and wears 5.5 size larger shoes. Subjects 6 and 20 are both of the same gender with only a 1 cm of height difference, are within 1 shoe size of each other and are only 5 years apart in age. However, both are identified with 100% accuracy with no pairwise misclassification. Furthermore, subject 18 is sometimes misclassified as subject 8 (14.4%), and subject 8 is misclassified as subject 18 (4.1%). However, these two subjects are of different genders who are also 35 years, 10 cm and 2 sizes apart in terms of age, height and shoe sizes. Therefore, while there may be some weak correlation between the subjects’ characteristics and the models’ inherent feature extraction, it cannot be used to reasonably explain the errors currently present. Furthermore, we explored whether the network structure used for identifying gait modes on capacitive SensFloor [9] could also be used for gender recognition. While the LSTM approach in SensFloor offered a significant reduction in the number of learnable parameters when compared to our approaches (<20,000 vs. 620,000>), it performed worse than all our tuned models with a classification accuracy of 0.685 for women and 0.753 for men. The structure was also tested for classifying identity and was unable to correctly identify individuals (classification accuracy below 0.5). This suggests that fair comparison can only be undertaken by comparing and optimizing algorithms on the same dataset. The algorithms were trained using a A100 GPU provided by Nvidia in Matlab 2021b. Classification on the test data was completed using an i7-8700 CPU. The top performing BLSTM identity model can output a classification in 0.5 ms, and the top performing CNN gender model can output a classification in 0.36 ms, enabling real-time classification on input data streams.

## 4. Conclusions

**Figure 10:**
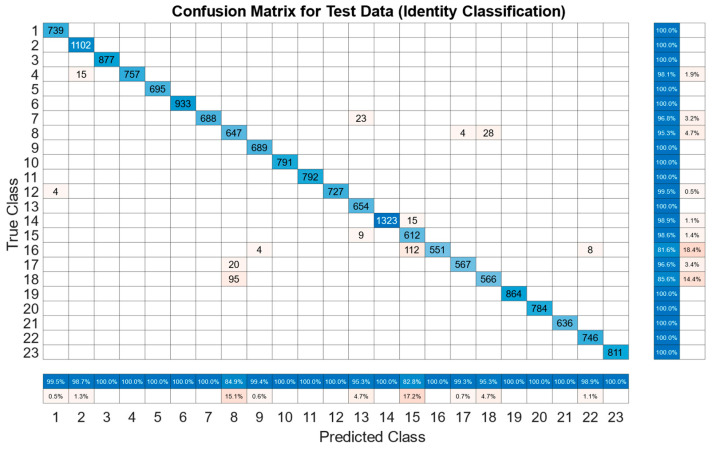
Identity Classification using Final BLSTM Model.

**Figure 11:**
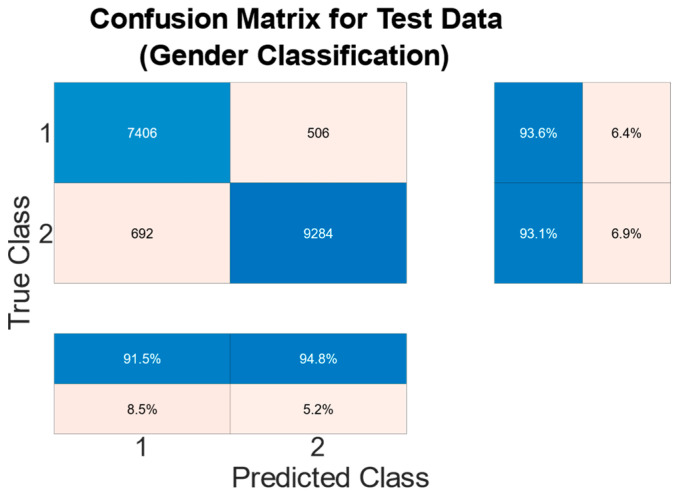
Gender Classification using Final CNN Model.

To the best knowledge of the authors, this work offers the first comparison of traditional, spatial and temporal neural networks for use in identity and gender classification for a capacitive sensing floor. We benchmarked five neural network architectures (CNN, MLP, LSTM, BLSTM and GRU) against SVM, which is a traditional classifier used for floor-based recognition. Accuracies of up to 98.12% were achieved for identity classification using the BLSTM structure as shown in Figure 10, and up to 93.3% for gender recognition using a CNN structure as shown in Figure 11. There may be scope for increasing the number of panels to allow multiple subjects to walk the floor at the same time. Future work should explore simultaneous multi-target localization and identity recognition. Furthermore, it is likely that this type of flooring may not be able to be deployed in all areas. Therefore, integration with other localization methods using different sensing modalities should be explored. If a subject has foot injury that causes significant changes to the gait pattern, CapLoc may not be able to identify the subject correctly. However, this also suggests that CapLoc could potentially identify gradual gait degradation that is often a precursor to frailty (leading to the risk of fall) and many neurological diseases that causes gait abnormality. This type of detection/prediction could be achieved in an automated manner and outside of the clinical setting by non-intrusively monitoring a user while they are going through their regular daily routine in their own home. This is another avenue of future research that we wish to investigate in the future. Intruder detection is a potential application of CapLoc and was not explored in this work. Such a task would require identifying unregistered class. One-class classifiers have shown promising performance with seismic sensing-based imposter identification [21], and they can be investigated in the future for intruder detection.

## Figures and Tables

**Table 1:** Floor-Based Privacy-Preserving Human Classification Approaches.

Source	Measurement Method	Classification Goal	Accuracy	Algorithm	Participants
Proposed	Capacitive Flooring	Identity/Gender	0.98/0.93	BLSTM/CNN	23
Bales et al. [39]	Underfloor Accelerometer	Gender	0.88	SVM	15
Anchal et al. [12]	Geophone	Gender	0.956	SVM	8
Qian et al. [44]	Pressure Sensors	Identity	0.92	Fisher LD	11
Mukhopadhyay et al. [21]	Geophone	Identity	0.92–0.98	SVM	8
Clemente et al. [45]	Seismometer	Identity	0.97	SVM	6
Miyoshi et al. [22]	Microphone	Identity	0.928	GMM	12
Shi et al. [36]	Capacitive (Triboelectric Sensor)	Identity	0.96	CNN	10
Shi et al. [37]	Capacitive (Triboelectric Sensor)	Identity	0.8567	CNN	20
Li et al. [38]	Capacitive (Triboelectric Sensor)	Identity	0.976	BLSTM	8
Pan et al. [19]	Geophone	Identity	0.9	SVM	10

**Table 2:** Neural Network Hyperparameter Values Used in the Final Tuned Identity Models.

Algorithm	Hyperparameter Range	Final Hyperparameter Values
**CNN**	Epsilon: [1 × 10^−8^, 1] Section depth: [1, 4]	Epsilon: 0.0388 Section depth: 4
	Filter size: [2, 7]	Filter size: 5 (5 × 5)
	Pooling: [1, 5]	Pooling: 1 (No pooling)
	Number of filters: 2^[3, 9] Fully connected layer neurons: 2^[5, 10]	Number of filters: 256 Fully connected layer neurons: 1024
**MLP**	Epsilon: [1 × 10^−8^, 1] Section depth: [1, 4]	Epsilon: 0.081 Section depth: 2
	Neurons: 2^[4, 10]	Neurons: 1024
	Dropout: [0, 0.4]	Dropout: 0.19
**LSTM**	Epsilon: [1 × 10^−8^, 1] Number of hidden units (1): 2^[4, 10]	Epsilon: 0.0013 Number of hidden units (1): 512
	Dropout (for hidden layer 1): [0, 0.4] Number of hidden units (2): 2^[4, 10]	Dropout (for hidden layer 1): 0.369 Number of hidden units (2): 256
	Dropout (for hidden layer 2): [0, 0.4]	Dropout (for hidden layer 2): 0.116
**BLSTM**	Epsilon: [1 × 10^−8^, 1] Number of hidden units (1): 2^[4, 10]	Epsilon: 0.00192 Number of hidden units (1): 1024
	Dropout (for hidden layer 1): [0, 0.4] Number of hidden units (2): 2^[4, 10]	Dropout (for hidden layer 1): 0.354 Number of hidden units (2): 64
	Dropout (for hidden layer 2): [0, 0.4]	Dropout (for hidden layer 2): 0.19
**GRU**	Epsilon: [1 × 10^−8^, 1] Number of hidden units (1): 2^[4, 10]	Epsilon: 0.00054 Number of hidden units (1): 1024
	Dropout (for hidden layer 1): [0, 0.4] Number of hidden units (2): 2^[4, 10]	Dropout (for hidden layer 1): 0.34 Number of hidden units (2): 128
	Dropout (for hidden layer 2): [0, 0.4]	Dropout (for hidden layer 2): 0.06

**Table 3:** Neural Network Hyperparameter Values Used in the Final Tuned Gender Models.

Algorithm	Hyperparameter Range	Final Hyperparameter Values
**CNN**	Epsilon: [1 × 10^−8^, 1] Section depth: [1, 4]	Epsilon: 0.0058 Section depth: 2
	Filter size: [2, 7]	Filter size: 5 (5 × 5)
	Pooling: [1, 5]	Pooling: 1 (No pooling)
	Number of filters: 2^[3, 9]Fully connected layer neurons: 2^[5, 10]	Number of filters: 128 Fully connected layer neurons: 256
**MLP**	Epsilon: [1 × 10^−8^, 1] Section depth: [1, 4]	Epsilon: 0.008 Section depth: 4
	Neurons: 2^[4, 10]	Neurons: 512
	Dropout: [0, 0.4]	Dropout: 0.047
**LSTM**	Epsilon: [1 × 10^−8^, 1] Number of hidden units (1): 2^[4, 10]	Epsilon: 0.03118 Number of hidden units (1): 1024
	Dropout (for hidden layer 1): [0, 0.4] Number of hidden units (2): 2^[4, 10]	Dropout (for hidden layer 1): 0.27 Number of hidden units (2): 32
	Dropout (for hidden layer 2): [0, 0.4]	Dropout (for hidden layer 2): 0.05
**BLSTM**	Epsilon: [1 × 10^−8^, 1] Number of hidden units (1): 2^[4, 10]	Epsilon: 0.00192 Number of hidden units (1): 256
	Dropout (for hidden layer 1): [0, 0.4] Number of hidden units (2): 2^[4, 10]	Dropout (for hidden layer 1): 0.38 Number of hidden units (2): 16
	Dropout (for hidden layer 2): [0, 0.4]	Dropout (for hidden layer 2): 0.18
**GRU**	Epsilon: [1 × 10^−8^, 1] Number of hidden units (1): 2^[4, 10]	Epsilon: 0.00391 Number of hidden units (1): 1024
	Dropout (for hidden layer 1): [0, 0.4] Number of hidden units (2): 2^[4, 10]	Dropout (for hidden layer 1): 0.36 Number of hidden units (2): 16
	Dropout (for hidden layer 2): [0, 0.4]	Dropout (for hidden layer 2): 0.12

**Table 4:** Test Performance of the Final Tuned Identity Models.

Algorithm	Precision	Recall	Accuracy	F1 Score	Total Learnable Parameters
**CNN**	0.92065	0.942478	0.936	0.931436	57,378,071
**MLP**	0.89125	0.917696	0.913	0.90428	1,279,399
**LSTM**	0.92165	0.944348	0.939	0.932861	2,253,591
**BLSTM**	0.9776	0.978696	0.9812	0.978148	11,120,023
**GRU**	0.88815	0.913348	0.906	0.900573	3,691,927
**SVM**	0.82615	0.863478	0.854	0.844402	129,930 × 200 *

* Total number of Support Vectors used by the 23 binary learners trained for the SVM.

**Table 5:** Test Performance of the Final Tuned Gender Models.

Algorithm	Precision	Recall	Accuracy	F1 Score	Total Learnable Parameters
**CNN**	0.9335	0.9315	0.933	0.932499	6,967,938
**MLP**	0.8885	0.8845	0.887	0.886495	892,306
**LSTM**	0.8945	0.889	0.890	0.891742	5,152,962
**BLSTM**	0.848	0.8495	0.839	0.848749	620,674
**GRU**	0.89	0.8845	0.884	0.887241	3,813,202
**SVM**	0.855	0.85	0.85	0.852493	15,053 × 200 *

* Total number of support vectors used by the single binary learner trained for the SVM.
